# Associations Between Systemic Diseases and Dental Pulp Diagnoses: A Retrospective Chart Study Using a Locally Deployed Large Language Model

**DOI:** 10.3390/dj14090575

**Published:** 2026-09-07

**Authors:** Yoshifumi Kobayashi, Shuying Jiang, Jia Huang, Tami Kim, Kathryn Roeder, Liyaa Chen, Nika S. Kobayashi, Daniel H. Fine, Emi Shimizu

**Affiliations:** 1 Department of Oral Biology, Rutgers School of Dental Medicine, Newark, NJ 07103, USA; kobayayo@rwjms.rutgers.edu (Y.K.); jh1707@rutgers.edu (J.H.); tk504@sdm.rutgers.edu (T.K.); kr808@sdm.rutgers.edu (K.R.); nk1134@sdm.rutgers.edu (N.S.K.); finedh@sdm.rutgers.edu (D.H.F.); 2 Academic Affairs, Rutgers School of Dental Medicine, Newark, NJ 07103, USA; jiangss@sdm.rutgers.edu; 3 Office of Information Technology, Rutgers, The State University of New Jersey, Newark, NJ 07101, USA; chenliy@oit.rutgers.edu

**Keywords:** dental pulp, endodontics, cancer, HIV, diabetes mellitus, chart study, LLM

## Abstract

**Objectives**: The objectives of this study were as follows: (1) statistical investigation of the association between systemic diseases and dental pulp condition; and (2) utilization of locally running large language models (LLMs) to handle a large number of dental charts written in natural languages, while protecting patients’ information. **Methods**: A total of 5820 endodontic charts, including healthy controls and patients with a history of cancer, HIV, type 1 diabetes mellitus (DM), and type 2 DM, were selected from 15,147 samples. Using a local LLM environment, the dental charts from each group were classified into six categories: normal pulp (NP); reversible pulpitis (RP); asymptomatic irreversible pulpitis (AIP); symptomatic irreversible pulpitis (SIP); pulp necrosis (PN); and “others”, including unclear diagnoses. The distributions of these diagnoses across groups were statistically analyzed. **Results**: The prompt for the local LLM was repeatedly refined by classifying 400 test cases, resulting in 97.0% agreement with the judgments of three human dentists. The refined prompt was then applied to classify 5820 charts, followed by statistical analyses. Using a generalized linear mixed-effects model (GLMM), the cancer-history group showed a significantly higher NP and a lower AIP proportion, whereas the type 2 DM group showed a significantly lower AIP and a higher SIP proportion. **Conclusions**: The findings in the cancer-history group could be attributable to a worsened oral environment in the patients and difficulties in diagnosing AIP. Meanwhile, the lower proportion of AIP and higher proportion of SIP in type 2 DM group may reflect pulpal inflammation associated with the disease. Additional attention may be warranted for caries’ prevention and treatment in patients with type 2 DM.

## 1. Introduction

### 1.1. Systemic Diseases and Dental Pulp Condition

The association of systemic factors of patients with their pulp condition has been gradually clarified in the past two decades. Researchers have found that diabetes mellitus (DM) negatively affects the success rate of root canal treatment (RCT) [1] and that DM, cardiovascular diseases, osteoporosis, HIV infection, or inflammatory bowel disease can influence or interfere with periapical tissue repair after RCT [2]. These studies have clearly shown the relationship between systemic factors and pulp condition. Furthermore, the other group reported the association between apical periodontitis prevalence and several systemic factors, including DM, irritable bowel disease, osteoporosis, renal disease, inherited coagulation disorders, and smoking [3]. This study more directly connected systemic diseases and pulp condition by measuring the prevalence of endodontic symptoms, not treatment outcomes, which is quite relevant from the pulp-biology perspective.

This type of pulp condition assessment has been largely focused on pulp stone formation, because pulp stone prevalence has a potential to be used to determine the presence of certain diseases. For example, pulp stone prevalence is significantly correlated with hypertension and type 2 DM [4], cardiovascular disorders [5,6,7,8], nephrolithiasis [9], or autoimmune diseases [10]. Besides such symptomatologic purposes to detect diseases, the effects of systemic diseases on dental pulp condition are increasingly investigated because the information is quite valuable when treating such patients in dental clinics [2].

In patients with a history of cancer, disease therapies, rather than the disease itself, including chemotherapy or radiotherapy, affect pulp condition. Radiotherapy for the face and neck significantly decreases pulp oxygen saturation, leading to lower pulp vitality after treatment [11]. Paclitaxel was reported to reduce the number of calcitonin gene-related peptide (CGRP)-positive nerve fibers in the dental pulp, affecting tertiary dentine formation in vivo [12]. Furthermore, systemic bacterial infections from teeth in patients suffering myelosuppression caused by chemotherapy have been reported [13,14].

The effects of HIV infection on the pulp condition have not been systematically reviewed so far. However, some reports have demonstrated clear characteristics of patients with HIV; for example, distinct kinds of chronic inflammatory cells, the absence of polymorphonuclear leucocytes, and widely dilated pulpal blood vessels were observed in the periapical histology sample of a patient with HIV [15]. Patients with HIV have a prolonged proinflammatory response in the periapical area after RCT [16]. They complain of more pain in diagnosis and experience prevalent hemorrhage during endodontic treatment, although no significant difference in the final outcome was observed at 24 months [17]. Earlier pain resolution appeared in the HIV-positive group 48 h after surgery, although other pain patterns were comparable to those observed in HIV-negative individuals [18].

The effects of DM on the dental pulp are known to be multifaceted [19,20,21], including altered inflammatory responses, modified immune cell function [22], compromised microvascular circulation, reduced tissue repair [23,24], reduced dental pulp cell viability [25,26,27], decreased melatonin levels [28], increased alkaline phosphatase levels [29], and significantly higher prevalence of apical periodontitis (AP) [30,31,32]. Furthermore, animal studies have shown that DM increases the inflammatory infiltration in AP, causing significant overexpression of TLR2 and TLR4 in the periapical region [33], hindering pulp healing [34,35], delaying tooth development, and impairing stem/progenitor-cell differentiation [36]. These changes in pulp conditions observed in human or animal models of DM highlight the significance of DM effects on pulp wellness, which may assist in the development of guidelines for the treatment of patients with DM.

### 1.2. Utilization of LLMs in Chart Interpretation or Classification

LLMs have been increasingly used in the medical/dental field, particularly in medical writing, education and training, diagnosis and treatment, and patient communication (reviewed in [37]).

Because LLMs are designed to “interpret” natural languages, chart analysis, classification, interpretation, or data extraction are the most suitable fields for LLM use. Many examples are found in medical research [38,39,40]. However, examples of LLM application in dental chart analysis/classification are limited. Chang’s group used the combination of GPT-J and RoBERTa for named entity recognition (NER) on periodontal notes [41]. The same group trained GPT-4 combined with RoBERTa to interpret periodontal diagnosis and extract data from them [42]. These studies excellently demonstrated the potential of LLMs in interpreting dental charts written in natural language.

In the present study, we statistically analyzed the association between pulp diagnosis and systemic diseases by using a locally running LLM for dental chart interpretation. The aim of this study was to infer the effects of each systemic disease on dental pulp condition and to make chart studies more researcher-friendly and effortless.

## 2. Materials and Methods

### 2.1. Dental Charts

All procedures associated with this study were reviewed by the Institutional Review Board (IRB) of Rutgers School of Dental Medicine (#Pro2018001075), in accordance with the Health Insurance Portability and Accountability Act of 1996 (HIPAA). We collected 15,147 dental charts, with current dental terminology (CDT) codes indicating endodontic procedures (D3310, D3320, and D3330) between May 2018 and October 2023, from the axiUm system of Rutgers School of Dental Medicine. All charts were handled by the staff of Information Systems and Technology (IST) Department and Office of Clinical Affairs, and patient information in those charts was de-identified, except for the tooth ID, sex, age during the service, medication and health records, and dentist’s note stating diagnosis and treatments. Medical records were classified using Microsoft Excel by IST members and lab members who have experience in our previous similar work [43]. All data containing chart number = patient ID, site number = tooth ID, notes = endodontic dental chart, sex, and age were changed to the .csv format when they were fed to the LLM.

### 2.2. Software and Operating Environment

The local LLM openai/gpt-oss-20b (OpenAI, 2025, San Francisco, CA, USA), the platform LM Studio 0.3.26 (lmstudio.ai, Elements Labs, 2025), Python 3.13.7 (python.org), and its libraries were downloaded to a Windows 11-operated laptop computer equipped with Intel^®^ Ultra 9 185H (2.30 GHz), 32 GB RAM, and 1 TB storage. The Python code (Appendix A) to feed diagnostic data to LM Studio was produced using Copilot (Microsoft) and was run in the IDLE environment (python.org).

LM Studio was loaded with openai/gpt-oss-20b and used in the developer mode controlled by the Python code shown in Appendix A. The settings were changed from the default to context length of 30k token, temperature of 0.01, and medium reasoning.

### 2.3. Statistical Analysis

In this study, 139 patients who had two or more medical records were excluded from the data analysis. A total of 2408 patients with 2967 teeth were included in the data analysis. Of these, 82% had one tooth, 14% had 2 teeth, 2.5% had 3 teeth, 1% had 4 teeth, and 0.29% had ≥5 teeth. The average number of teeth for each patient was 1.23.

The cases were divided into categories using Excel (Microsoft) and analyzed with chi-squared tests and logistic regression using the statistic software JASP Version 0.96.0 (JASP Team (2026)) and IBM SPSS Statistics Version 30.0.0.0.

Because some patients contributed more than one tooth, a generalized linear mixed-effects model (GLMM) with a logit link and binomial distribution was used to account for the correlation of teeth within the same patient. NP (yes/no), AIP (yes/no), SIP (yes/no), and PN (yes/no) were the binary dependent variables. Medical records (healthy, cancer, HIV, type 1 DM, and type 2 DM), sex (F/M), and age (young, 19–39; middle, 40–59; and old, ≥60) were included as fixed effects. Healthy participants, females, and young age group were used as the reference categories for medical records, sex, and ages, respectively. Patient ID was included as a random intercept to account for within-patient clustering. Results are presented as odds ratios (ORs) with 95% confidence intervals (CIs). A two-sided *p*-value < 0.05 was considered statistically significant.

### 2.4. Classification of the Purposes of RCT on NP

The purposes of RCT on NP were classified based on the terminology that was repeatedly used in the charts analyzed in this project. RCT for restoration indicates cases that require RCT to set post and core for building a large restoration on a tooth. Prophylactic RCT indicates cases in which a dentist judged that the pulp would develop an SIP or NP without RCT although the pulp seemed normal.

## 3. Results

### 3.1. Prompt Refinement to Instruct the Local LLM

The prompt to instruct the local LLM (Appendix B) was repeatedly refined through the evaluation of the test case of 400 endodontic charts. After >30 trials, the prompt resulted in 97% consistency with three human dentists from Rutgers School of Dental Medicine (Table 1). The differences of judgement between the dentists and our LLM at the final round of the 400 case trials are shown in Appendix C.

Furthermore, to evaluate the functionality of the refined prompt, 400 independently chosen charts were classified using the prompt (Table 2). In this trial, the consistency with human dentists was 95.3%, and Cohen’s kappa was 0.926 (standard error = 0.017; 95% CI, 0.893–0.958).

### 3.2. Classification of Endodontic Charts

By checking the medical history, the collected 15,147 charts were categorized into the following cohorts: healthy control (without any medical history or medication (*n* = 3904)), cancer history (*n* = 453), HIV (*n* = 431), type 1 DM (*n* = 59), and type 2 DM (*n* = 973) groups. These diseases were selected due to their relatively high frequency in the charts collected and their association with our previous work [43]. The charts from patients who had more than two medical records were excluded. Using the local LLM, charts from each group were classified into six categories in accordance with the terminology released by the American Association of Endodontists [44]: normal pulp (NP); reversible pulpitis (RP); asymptomatic irreversible pulpitis (AIP); symptomatic irreversible pulpitis (SIP); pulp necrosis (PN); and “others”, including previously initiated or treated and unclear diagnoses (Table 3). To analyze correlations with age, patients were categorized into three age groups (18–39, 40–59, and ≥60), as previously described [43] (Table 3). The patients were also categorized according to sex (Table 3).

### 3.3. Generalized Lenear Mixed-Effects Model (GLMM)

Because the collected charts included some patients contributing data on multiple teeth (see Materials and Methods, Section 2.3), a GLMM was used to analyze the association between systemic diseases and dental pulp condition, while separating the influences of age and sex from those of medical records (Table 4). Here, the data analyzed using RP distribution were omitted because the sample size was too small to yield a meaningful result. Interestingly, the cancer group demonstrated significantly higher NP (OR: 1.61) and lower AIP (OR: 0.53) than the healthy control group, whereas no significant differences in SIP and PN were observed between the two groups. The type 2 DM group demonstrated significantly lower AIP (OR: 0.67) and higher SIP (OR: 1.30), and the type 1 DM group showed similar tendency (AIP: OR, 0.32; SIP: OR, 1.54). Furthermore, the three age groups demonstrated significant differences in NP, SIP, and PN. Similarly, the male category demonstrated significant changes with ORs of 0.69 for SIP and 1.38 for PN (Table 4). These data indicate the association between cancer or DM and pulp diagnosis, as well as the influences of age and sex. Simultaneously, the correlation with sex, which has been suggested in previous reports, was rediscovered in this analysis (see discussion part).

## 4. Discussion

### 4.1. Association Between Disease History and Pulp Condition

In this study, our analyses predicted the next associations between systemic diseases and pulp diagnoses.

1. Along with aging, NP and PN increase, whereas SIP decreases.
2. Males exhibited more PN and less SIP than females.
3. In the type 2 DM group, AIP was lower and SIP was higher. A similar tendency was observed in the type 1 DM group.
4. In the cancer-history group, NP was higher and AIP was lower.

The detailed discussion regarding each finding, using current knowledge related to them, continues below.

#### 4.1.1. Age

In a pulp sensibility test, the pain intensity in the older group was slightly lower compared to the younger group [45]. The number of blood vessels and nerves in the coronal pulp was lower in the aged group [46]. Furthermore, GAP43 and p75NTR, highly expressed non-myelinating Schwann cell markers, are significantly decreased in the teeth of older adults [47]. The density of myelinated axons in the dental pulp of older adults is significantly reduced [47]. These observations are consistent with the hypothesis that the attenuation of neural activities in the pulp of older adults prevents their sensing of bacterial infections in the early stage. Moreover, a transcriptomic analysis of dental pulp demonstrated that gene expressions of cell and tissue differentiation, development, and proliferation and of the immune, lymphatic, and hematologic systems are not active in the older group compared to those observed in the younger group [48]. Senescence impairs the regenerative and repair capacity of the dental pulp under inflammatory conditions [49]. Such decreases in pulp function could promote a rapid progression of necrosis after initial bacterial infection, resulting in a decrease in SIP and an increase in PN in the older group.

Regarding the increased NP in the older group, to elucidate this phenomenon, the purposes of RCT for NP in three age groups (18–39, 40–59, and ≥60) were categorized into six subgroups: restorative, prophylactic, no RCT performed, NP but apical periodontitis (AP), making abutment for bridge or denture attachment, and others (Table 5). In this process, the charts of returning patients were excluded to restrict one tooth per patient. The chi-square test analyzing the obtained distribution revealed that elective RCT with restorative purposes significantly increased in the older group, suggesting that the increase in restorative RCT is possibly the main reason for the increase in NP in the older group. In older individuals, tooth structure becomes brittle and weak [50], and the requirement of restorative procedure, including post and core, along with RCT, may naturally increase.

#### 4.1.2. Sex

More PN and less SIP diagnoses have been reported among males [51,52], while one study showed no difference in PN prevalence [53]. One of the works in the literature explained this phenomenon with the hypothesis that females are more concerned about their oral health [52]. However, we do not proceed with the discussion regarding the sex difference shown here, because the study sample is limited size-wise and geo-culturally.

#### 4.1.3. Type 2 DM

The higher SIP and lower AIP diagnoses in the type 2 DM group could be associated with proinflammatory conditions often observed in the dental pulp of patients with this disease [54,55] (reviewed in [21,56,57]). In the dental pulp of patients with DM, the inflammatory cytokines IL1β, IL6, IL17, and TNF-α are significantly increased [58]. These cytokines can sensitize nociceptive nerves, enhancing pain perception in the dental pulp of patients with DM [59].

Additionally, it is noteworthy that higher SIP diagnoses in younger patients with type 2 DM patients were reported previously [43,60], whereas PN diagnosis is dominant, rather than SIP, in older type 2 diabetes groups in those same reports. This disparity could be explained by the high prevalence of type 2 DM in the older population than in the younger population [61] (Appendix D). This sampling bias of patients with type 2 DM toward the older group might have “masked” the dominancy of SIP by the dominancy of PN in older individuals, as is discussed in Section 4.1.1. above. Nevertheless, a stronger control with confounding factors regarding the type 2 DM group in our study will be required to increase the accuracy of the aforementioned hypotheses.

#### 4.1.4. Cancer

To infer the cause of increasing NP and decreasing AIP in the cancer-history group, the purposes of RCT for NP in the group were categorized into six subgroups, as done in Section 4.1.1., the discussion for the correlation between age and pulp condition (Table 6). The chi-square test analyzing the obtained distribution revealed a relative increase in RCT with restorative purpose and a significant decrease in RCT with prophylactic purpose in the cancer group, suggesting that the increase in restorative RCT could be associated with the increase in NP in the cancer group.

Restorative RCT is performed mainly when only insufficient tooth structure is left to support the coronal restoration [62]. Meanwhile, xerostomia caused by damaged salivary glands due to head-and-neck radiation or chemotherapy for patients with cancer is broadly known to make an oral environment susceptible to caries [63,64,65,66]. Therefore, the next hypothesis could be derived from observation #4: Xerostomia caused by cancer treatments exacerbates the oral environment in cancer survivors, leading to more frequent restorative RCT, which increased the number of NP in cancer survivors compared with that observed in the healthy group. Further, such exacerbated oral environment in cancer survivors could rapidly worsen caries condition and lead to significant decrease in “prophylactic” stage of caries, as shown in the aforementioned chi-square analysis. Second, the aforementioned increase in restorative RCT could be associated with the reduction in AIP frequency in cancer survivors, because there are some difficulties when distinguishing between NP and AIP on diagnosis. For example, if there is a large decay adjacent to the pulp space but the patient does not report pain and the cold test result is ambiguous, the pulp can be diagnosed as AIP, or the endodontist might recommend restorative RCT as a treatment for NP tooth [44,67]. Another possible hypothesis for the decreased AIP is that chemotherapies for cancer attenuated the immune system in the pulp tissue of patients with cancer, losing resistance to the rapid progression from AIP to SIP or PN during pulp infection, reducing AIP in cancer survivors. However, if it is the case, both SIP and PN should increase in cancer survivors, which is not clear in this study (Table 4).

However, the above discussions have a limitation caused by the absence of detailed medical records for the cancer-history group. Obtaining and analyzing information regarding salivary gland conditions or chemotherapy for the group will add more accuracy to the discussions.

### 4.2. Local LLM for Chart Study

In this study, a local LLM, openai/gpt-oss-20b, was used to interpret dental charts and classify them according to patients’ pulp condition. This local LLM was used because it is suitable to prevent the bleaching of patient information or the machine learning of patient information by AIs owned by some corporations [68,69,70]. Furthermore, unlike LLMs on the servers employed by corporations or universities, the results of machine learning in a local LLM are temporal—that is, once the working computer is turned off, the acquired weights and parameters in the LLM system optimized through training by each user are lost and go back to the default status, except for some settings. In that regard, employing a local LLM in chart studies implies choosing confidentiality over productivity. It should be particularly noted that the present chart study is based on such selection, weighing on confidentiality. In contrast, the processing speed of chart interpretation (approximately one minute per dental chart) has room for improvement, which may need a desktop computer equipped with a high-end GPU or a 1-bit LLM bonsai-8B, utilizing a new technology to reduce the size of LLMs (PrismML, Pasadena, CA, USA).

Another point to be noted when using a local LLM due to the limited machine learning is that prompt engineering becomes the main task in chart studies. In the present study, our prompt was revised >30 times to achieve 97% consistency with human dentists (Results, Section 3.1); we used several techniques, including “chain-of-thought”, in which an LLM is instructed to “think step-by-step” by feeding with multiple simple tasks instead of a few complex ones (Appendix B). Especially, repetition of similar thinking tasks for multiple cycles significantly reduced misinterpretation by the LLM in the present study (Appendix B). Furthermore, a parameter called “temperature”, which typically ranges from 0.0 to 2.0, reflecting the randomness of LLM inference, was set to 0.01 to limit the uncertainty of LLM behavior (Appendix A).

In the entire dental field, a substantial number of trials utilizing LLMs have been reported (reviewed in [71,72,73,74,75,76]). Those trials span a broad range of dentistry fields, including diagnostics, therapeutic management, patient management, and dental education. Furthermore, the usage of LLMs has started across many clinical departments, including pediatric dentistry, prosthodontics, orthodontics, periodontics, endodontics, and forensic odontology [71,72]. Within those reports, “hallucination” was claimed as the largest barrier to pursuing accuracy in each project. “Hallucination” is a plausible but incorrect response from AI, which can lead to misdiagnoses or inaccurate medical advice [77]. To address this issue, developers and users have adopted various technologies, such as the improvement of learning algorithms; increasing the size and quality of training datasets, prompt engineering; and especially, new methodologies for the validation of AI’s responses, including retrieval-augmented generation using external information and human-in-the-loop feedback [71]. With these improvements, in dental diagnostic field, DeepSeek-R1 has achieved an accuracy of 83.8% in the dental diagnostic field [78]; however, in a literature review covering overall LLM usage in dental diagnoses, accuracies reported so far are exhibiting a range as broad as 33.3–92.5% [79]. This indicates that validation by humans and comparison with external information are still needed for the safe use of LLMs in dental clinics and research fields. In the current study, extensive prompt refining increased the consistency with human dentists to >95%. However, compared with LLM usages in other dental fields, there is a possibility that our relatively high score is due to the specificity of the given task, focusing only on interpretation of natural language.

Nevertheless, the combination of the local LLM openai/gpt-oss-20b and LM Studio was sufficient for chart study conducted by laypersons regarding computers, except for the speed. Openai/gpt-oss-20b is released by OpenAI itself, not by a third-party modifier, and complies with the Apache 2.0 license, allowing for free local hosting, modifications, and commercial use. LM Studio provides a graphical interface for easy use by researchers who are not coding experts. Both can be downloaded from their official sites without expense. The Python program to feed each dental chart was produced by having conversations with a commercial AI, Copilot (Microsoft) (Appendix A), owing to the user-friendliness of openai/gpt-oss-20b and LM Studio.

### 4.3. Limitations

This chart study analyzed data from the dental charts written between May 2018 and October 2023 at Rutgers School of Dental Medicine. Accordingly, the data contain notes written by a substantial number of students and professors, which naturally introduce variability regarding the documentation style, diagnostic terminology, and clinical judgments. It should be noted that the results of analyses in the present study would be affected by such variabilities.

The next limitation is that more than half of the charts were classified as “others”. Because this phenomenon happened in the classifications performed by both the LLM and human dentists (Table 1 and Table 2), it would be reasonable to assume that the phenomenon was related to the high frequency of returning patients or the difficulty of classifying chart notes written in natural language. To assess if the “others” charts have some bias on patients’ medical records, a chi-square test was performed between diagnosed and “others” charts regarding their medical records (Table 7), following the exclusion of returning patients. The proportions of charts categorized as “others” relative to diagnosed ones showed significant shifts in patents with HIV, cancer survivors, and type 2 DM group. Regarding the HIV group, because returning patients were excluded, the significant increase in the ratio can be related to the difficulties in classifying their charts. This difficulty could be associated with the tendency that up to 50–80% of patients with HIV are also accompanied by other oral manifestations such as candidiasis and periodontitis [80,81]. Such multi-oral manifestations may complicate the symptoms/diagnosis of patients with HIV. Similarly, the changes in the “others” ratio observed in cancer group and type 2 DM group could be explained by the frequency of difficult dental diagnoses in each group. Namely, cancer survivors frequently have periodontitis-related complications (~65%) [82], as seen in HIV patients, which can be associated with the increase in “others”. In contrast, type 2 DM patients are prone to inflammatory pulp condition, as discussed in Section 4.1.3, which might lead to a severer pain to be diagnosed more explicitly as SIP. Nevertheless, the changes in the “others” ratio in the above groups poses the possibility of bias in the distribution of samples analyzed in this study.

Another limitation is that detailed health information of patents is not available in the charts used in this study. Even the healthy group may contain undocumented factors, such as smoking or other diseases. In particular, the cancer group may contain a broad spectrum of treatment history, including the regions of radiation and the types of chemotherapy, which affect patients’ oral environment differently. Therefore, the results obtained in the current study should be interpreted with caution. To address the aforementioned issues, future studies should prospectively collect patient information in each health condition.

## 5. Conclusions

In this study, electronic endodontic charts described in natural language in Rutgers School of Dental Medicine were classified into six pulp diagnosis categories, namely NP, RP, AIP, SIP, PN, and others, using a locally running LLM. The associations between the distribution of these pulp diagnoses and patients’ health records regarding systemic diseases, including cancer, HIV, type 1 DM, and type 2 DM, were statistically analyzed. The results showed the following: (1) with increasing age, SIP decreased, whereas NP and PN increased; (2) males had a higher proportion of PN and a lower proportion of SIP than females; (3) patients with type 2 DM had lower AIP and higher SIP; and (4) patients with a history of cancer had higher NP and lower AIP. All four statistical trends were explainable with the biological characteristics of the respective disease, age, or sex groups or were consistent with previously reported findings in endodontic patients. Notably, the GLMM analysis suggested that the apparent (not absolute) increase in PN among patients with type 2 DM could be attributable to the larger distribution of type 2 DM in older age groups. These findings highlight the importance of distinguishing individual factors in epidemiologic analyses, as well as the need for special attention to patients with systemic diseases. In particular, regarding type 2 DM patients and cancer survivors, additional preventive attention may be warranted to prevent caries’ formation, due to the possibility of rapid progression to pulpitis.

The local LLM met the requirements for chart studies emphasizing patient information protection, although its processing speed requires further improvement. With appropriate support for data processing and analysis, locally running LLMs may substantially reduce the extensive human effort required for retrospective chart classification.

## Figures and Tables

**Table 1:** Difference in interpretation between human dentists and the local LLM at the final round of the 400 case trials.

	Dentists	LLM	The LLM Interpreted As…	Changes	Totaled Difference
**NP**	16	15	1 AIP and 1 others	+1 − 2	−1
**RP**	4	3	1 others	−1	−1
**AIP**	26	27	1 others	+2 − 1	+1
**SIP**	78	82		+4	+4
**PN**	65	65	1 others	+1 − 1	0
**Others**	211	208	1 NP, 1 AIP, 4 SIPs, and 1 others	+4 − 7	−3
**Total**	400	400		+12 − 12	0

**Table 2:** Validation of the refined prompt using 400 independently chosen charts.

	Dentists	LLM	True Positive	True Negative	False Positive	False Negative	Sensitivity	Specificity	Precision	F1 Score
**Total**	400	400								
**NP**	13	14	11	384	2	3	0.786	0.995	0.846	0.915
**RP**	8	10	8	390	2	0	1.000	0.995	0.800	0.887
**AIP**	16	18	16	382	2	0	1.000	0.995	0.889	0.939
**SIP**	64	67	63	332	4	1	0.984	0.988	0.940	0.964
**PN**	75	82	75	318	7	0	1.000	0.979	0.915	0.945
**Others**	223	210	208	175	2	15	0.933	0.989	0.991	0.990

**Table 3:** Distribution of five endodontic diagnoses within the indicated categories. The shaded cells in the table are used to distinguish the content corresponding to (a) medical record, (b) age, and (c) sex. All headings are indicated in bold.

**(a) Medical record**														
			**NP**		**RP**		**AIP**		**SIP**		**PN**		**Others**	
		**Total**	***n***	**%**	***n***	**%**	***n***	**%**	***n***	**%**	***n***	**%**	***n***	**%**
**Medical Record**	**Healthy**	3904	125	3.2	47	1.2	234	6.0	855	21.9	734	18.8	1909	48.9
	**Cancer**	453	44	9.7	5	1.1	16	3.5	71	15.7	103	22.7	214	47.2
	**HIV**	431	16	3.7	7	1.6	19	4.4	58	13.5	74	17.2	257	59.6
	**Type 1 DB**	59	2	3.4	0	0.0	1	1.7	11	18.6	10	16.9	35	59.3
	**Type 2 DB**	973	60	6.2	8	0.8	44	4.5	201	20.7	222	22.8	438	45.0
**(b) Age: Patients’ ages were categorized into three groups (18–39, 40–59, and ≥60).**														
			**NP**		**RP**		**AIP**		**SIP**		**PN**		**Others**	
		**Total**	***n***	**%**	***n***	**%**	***n***	**%**	***n***	**%**	***n***	**%**	***n***	**%**
**Age Group**	**18–39**	2789	70	2.5	30	1.1	161	5.8	703	25.2	507	18.2	1318	47.3
	**40–59**	1959	83	4.2	26	1.3	107	5.5	371	18.9	382	19.5	990	50.5
	**≥60**	1072	94	8.8	11	1.0	46	4.3	122	11.4	254	23.7	545	50.8
**(c) Sex**														
			**NP**		**RP**		**AIP**		**SIP**		**PN**		**Others**	
		**Total**	***n***	**%**	***n***	**%**	***n***	**%**	***n***	**%**	***n***	**%**	***n***	**%**
**Sex**	**Female**	3317	134	4.0	39	1.2	178	5.4	771	23.2	605	18.2	1590	47.9
	**Male**	2503	113	4.5	28	1.1	136	5.4	425	17.0	538	21.5	1263	50.5

**Table 4:** GLMM treats patients’ age and sex as independent factors. CI, confidence interval. Significant differences (*p* < 0.05) are indicated by yellow highlights. The shaded cells in the table are used to distinguish the content corresponding to NP, AIP, SIP, and PN. All headings are indicated in bold.

**NP**					**AIP**				
		**Odds Ratio**	**95% CI**	***p***			**Odds Ratio**	**95% CI**	***p***
**Medical Record**	**Healthy**	Reference			**Medical Record**	**Healthy**	Reference		
	**Cancer**	1.61	1.05–2.47	0.028		**Cancer**	0.53	0.29–0.97	0.040
	**HIV**	1.02	0.58–1.77	0.954		**HIV**	0.88	0.51–1.53	0.656
	**Type 1 DB**	0.94	0.22–2.93	0.930		**Type 1 DB**	0.32	0.04–2.51	0.276
	**Type 2 DB**	1.04	0.77–1.50	0.817		**Type 2 DB**	0.67	0.45–0.99	0.048
**Sex**	**Female**	Reference			**Sex**	**Female**	Reference		
	**Male**	1.05	0.81–1.37	0.698		**Male**	1.07	0.82–1.38	0.632
**Age Group**	**18–39**	Reference			**Age Group**	**18–39**	Reference		
	**40–59**	1.51	1.09–2.10	0.014		**40–59**	1.20	0.89–1.61	0.232
	**≥60**	3.00	2.04–4.41	<0.001		**≥60**	1.12	0.72–1.72	0.620
**SIP**					**PN**				
		**Odds Ratio**	**95% CI**	***p***			**Odds Ratio**	**95% CI**	***p***
**Medical Record**	**Healthy**	Reference			**Medical Record**	**Healthy**	Reference		
	**Cancer**	1.06	0.74–1.52	0.746		**Cancer**	0.93	0.67–1.30	0.675
	**HIV**	0.96	0.66–1.40	0.835		**HIV**	1.03	0.72–1.45	0.887
	**Type 1 DB**	1.54	0.63–3.78	0.343		**Type 1 DB**	1.07	0.44–2.56	0.887
	**Type 2 DB**	1.30	1.02–1.67	0.038		**Type 2 DB**	0.95	0.75–1.20	0.661
**Sex**	**Female**	Reference			**Sex**	**Female**	Reference		
	**Male**	0.69	0.58–0.81	<0.001		**Male**	1.38	1.17–1.62	<0.001
**Age Group**	**18–39**	Reference			**Age Group**	**18–39**	Reference		
	**40–59**	0.64	0.53–0.78	<0.001		**40–59**	1.23	1.02–1.50	0.034
	**≥60**	0.30	0.22–0.40	<0.001		**≥60**	1.78	1.37–2.31	<0.001

**Table 5:** Chi-square test analyzing the distribution of RCT purposes in patients with normal pulp from three age groups. Significant differences (*p* < 0.05) are indicated by yellow highlights.

		Restorative			Prophylactic			No RCT			NP but AP			Abutment			Others		
Age Group	Total	*n*	%	*p*	*n*	%	*p*	*n*	%	*p*	*n*	%	*p*	*n*	%	*p*	*n*	%	*p*
**18–39**	60	3	5.0	<0.001	16	26.7	0.002	19	31.7	0.008	10	16.7	0.168	1	1.7	0.077	11	18.3	0.663
**40–59**	78	23	29.5		11	14.1		18	23.1		5	6.4		5	6.4		16	20.5	
**≥60**	75	31	41.3		3	4.0		6	8.0		7	9.3		9	12.0		19	25.3	
**Total**	213	57	26.8		30	14.1		43	20.2		22	10.3		15	7.0		46	21.6	

**Table 6:** Chi-square test analyzing the distribution of RCT purposes in patients with normal pulps from the healthy control or cancer group. Significant difference (*p* < 0.05) is indicated by yellow highlight.

		Restorative			Prophylactic			No RCT			NP but AP			Abutment			Others		
Medical Record	Total	*n*	%	*p*	*n*	%	*p*	*n*	%	*p*	*n*	%	*p*	*N*	%	*p*	*n*	%	*p*
**Healthy**	109	21	19.3	0.146	24	22.0	0.039	25	22.9	0.652	13	11.9	0.812	5	4.6	0.844	21	19.3	0.558
**Cancer**	37	12	32.4		2	5.4		7	18.9		5	13.5		2	5.4		9	24.3	
**Total**	146	33	22.6		26	17.8		32	21.9		18	12.3		7	4.8		30	20.5	

**Table 7:** Chi-square analyses of charts diagnosed as “others” regarding the medical records of the patients. Significant differences (*p* < 0.05) are indicated by yellow highlight.

	Diagnosed		Others		Total	*p*
Medical Record	*n*	%	*n*	%		
**Healthy**	1621	67.3	1790	65.0	3411	0.301
**Cancer**	180	7.5	256	9.3	436	0.025
**HIV**	155	6.4	278	10.1	433	<0.001
**Type 1 DM**	21	0.9	30	1.1	51	0.434
**Type 2 DM**	431	17.9	401	14.6	832	0.003
**Total**	2408	100.0	2755	100.0	5163	

## Data Availability

The original contributions presented in this study are included in the article. Further inquiries can be directed to the corresponding author under the restrictions to protect patients’ information.

## Abbreviations

The following abbreviations are used in this manuscript:

LLM	Large language model
DM	Diabetes mellitus
AP	Apical periodontitis
RCT	Root canal treatment
NP	Normal pulp
RP	Reversible pulpitis
AIP	Asymptomatic irreversible pulpitis
SIP	Symptomatic irreversible pulpitis
PN	Pulp necrosis
GLMM	Generalized linear mixed-effects model

## Appendix A

The following is the Python code to feed chart data individually, and the prompt for instructing LLM, into LM studio. Yellow highlights indicate adjustable parts. The author recommends checking white characters in the code below to prevent hidden code injection before you copy-and-paste it. Make sure to keep the indentation of each line.

** **

import pandas as pd

import requests

import json

** **

# Your CSV file name

csv_file_path = r"C:\...\Your Chart File.csv"

# LM Studio API endpoint (default)

api_url = "http://127.0.0.1:1234/v1/chat/completions"

** **

# Headers for the API request

headers = {

  "Content-Type": "application/json"

}

** **

# Read the CSV file into a pandas DataFrame

try:

  df = pd.read_csv(csv_file_path)

except FileNotFoundError:

  print(f"Error: The file ‘{csv_file_path}’ was not found.")

  raise SystemExit(1)

** **

# Helper: detect data-format-related errors in LLM content

def content_indicates_format_error(text: str) -> bool:

  if not isinstance(text, str):

    return True

  t = text.lower()

  keywords = [

    "data format error",

    "invalid format",

    "malformed",

    "unable to parse",

    "missing required",

    "bad schema",

    "wrong format",

    "incorrect format"

  ]

  return any(k in t for k in keywords)

** **

# Iterate through each row of the DataFrame

for index, row in df.iterrows():

  # Construct the prompt for the LLM based on the current row

  # Adjust this line based on your CSV columns and desired prompt structure

  prompt = f"…Your Prompt Without Line Breaks…"

** **

  # Optional: Format the prompt to include column names for clarity

  # prompt = f"Analyze the following data point: {‘, ‘.join([f’{col}: {val}’ for col, val in row.items()])}. Provide insights."

** **

  # Create the data payload for the API request

  payload = {

    "model": "openai/gpt-oss-20b", # Replace with the model loaded in LM Studio

    "messages": [

      {"role": "system", "content": "Read texts extremely carefully without missing any single word, using the information, choose the most accurate answer by strictly obeying the given every single rule."},

      {"role": "user", "content": prompt}

    ],

    "temperature": 0.01

  }

** **

  print(f"Processing row {index + 1}...")

** **

  # Send the request to the LM Studio API

  try:

    response = requests.post(api_url, headers = headers, data = json.dumps(payload), timeout = 300)

    response.raise_for_status() # Raise an exception for bad status codes (4xx or 5xx)

** **

    # Parse and print the LLM’s response

    llm_response = response.json()

 except (requests.exceptions.RequestException, ValueError) as e:

    print(f"HTTP/JSON error for row {index + 1}: {e}. Skipping this row.")

    print("-" * 50)

    continue # Skip this row and continue

** **

  # If the API returns an error object, skip the row

  if isinstance(llm_response, dict) and "error" in llm_response:

    err_msg = llm_response.get("error", {}).get("message", "Unknown error")

    print(f"LLM reported an error for row {index + 1}: {err_msg}. Skipping this row.")

    print("-" * 50)

    continue

** **

  # Safely extract content

  try:

    content = llm_response["choices"][0]["message"]["content"]

  except (KeyError, IndexError, TypeError) as e:

    print(f"Unexpected response shape for row {index + 1}: {e}. Skipping this row.")

    print("-" * 50)

    continue

** **

  # Detect format issues in the LLM’s output and skip if found

  if content_indicates_format_error(content):

    print(f"Data format issue detected in row {index + 1}. Skipping this row.")

    print("-" * 50)

    continue

** **

  # Otherwise, print the successful result

  print(f"Response for row {index + 1}:\n")

  print(content)

  print("-" * 50)

## Appendix B

This appendix contains the prompt to interpret dental notes and classify them to each endodontic diagnosis. This prompt needs to be inserted in Python code, such as the one shown in Appendix A, after removing all line breaks. The author recommends checking white characters in the prompt below to prevent hidden prompt injection before you copy-and-paste it. The length of the prompt could not be longer than the present size, because making it longer would cause hallucination and memory overflow in our system. “Note” was inserted due to the next reason. The endodontic diagnosis “SIP” was introduced in 2009 by the American Association of Endodontists, so that the dentists received education before the year or outside of the United States tend to use “IP” or “symptomatic pulpitis” instead.

Uploaded data array contains Chart number= patient ID, Site number = tooth ID, Notes = endodontic dental chart, gender, age. Using the data from this row: {row.to_json()}, classify the pulp condition of only the tooth annotated by the Site number, while carefully excluding other teeth’s information, into only one of the next categories; Normal pulp (NP), reversible pulpitis (RP), asymptomatic irreversible pulpitis (AIP), symptomatic irreversible pulpitis (SIP), Pulp necrosis (PN), and the others. ‘The others’ includes any previous treatments and no clear diagnosis. Never use assumption for this classification but strictly depend on clear statements in the chart.

During the classification procedure, start from step (I) below, with paying attention to the points (V). Prioritize the result of step (I) for the classification. If step (I) does not work, go to step (II). If step (II) does not work, go to step (III). If step (III) alone does not work, use the information written in (IV). If all above strategies did not work, just classify the tooth as ‘the others’. Output each classification and a brief reasoning to the next of each original patient ID, tooth ID, gender, age, in a single row. After the classification of all patients’ teeth, combine all outputs to a single table.

(I) Extensively search for an explicit diagnostic statement, normally written in the ‘A;’ area, ‘Dx’ area, and ‘diagnosis’ area of the chart. If it is found, use it for tooth classification and finish the classification. Diagnosis for periapical regions should be only used as a check point of PN. If there are two pulp diagnoses written for one tooth without the final decision, prioritize the statement in the ‘pulp diagnosis’ area. If still two diagnoses remain, carefully choose one of the two diagnoses using the strategy (II)-(IV) below. If enough information is not found, classify the tooth as ‘the others’ and finish the classification. Note: Mere ‘Irreversible pulpitis’ (IP) and ‘symptomatic pulpitis’ should be classified as SIP.

(II) Classify previously treated tooth first. If the chart contains expressions shown in the following A or B, classify the tooth as ‘the others’ and finish the classification. A. Actively search for the words indicating previous treatments, such as ‘previously initiated’, ‘prev initiated’, ‘PI’, ‘previously tx’, ‘continuation of treatment’, ‘to complete my treatment’, ‘initial RCT was done’, ‘following initial RCT’, or ‘changes after last visit’. B. Carefully check the existence of next temporary materials applied, not on the current visit, but on the previous visit. IRM (intermediate restorative material), cavit, or cotton.

(III) Judge from tooth test results. If none of these test data was shown in the chart, classify the tooth as ‘the others’.

A. Check caries or decay. 1. no caries or decay, 2. some caries or decay, 3. Deep (= close to the pulp) caries or decay, 4. Pulp exposure.
B. Check pain. 1. no pain, 2. Some pain, 3. Lingering pain, spontaneous pain.
C. Check cold test. 1. (-), 2. (+), 3. (+++).
D. Electric pulp test. 1. (-), 2. (+).
E. Check periapical radiolucency. 1.(-), 2. (+).
F. Check the existence of next temporary materials applied, not on the current visit, but on the previous visit. IRM (intermediate restorative material), cavit, or cotton.
1. No, 2. Yes.
F = 2 means previously treated root.
A = 4 means AIP or SIP or PN.
NP should show (A = 1 or 2) and B = 1 and (C = 2 or D = 2).
RP should show A = 2 and B = 2 and (C = 2 or D = 2).
AIP should show (A = 3 or 4) and B = 1 and (C = 2 or D = 2).
SIP should show (A = 3 or 4) and B = 3 and C = 3.
PN should show ((C = 1 or D = 1) and E = 2) and F = 1.
(IV) Judge from key information in the chart.
A. Conditions of NP:
1. ‘Elective endo’ or ‘intentional endo’, or RCT with restorative purpose in a chart indicates a prophylactic RCT for a tooth with normal pulp.
2. Conversely, the pulp treated with other than ‘elective endo’ or ‘intentional endo’, or RCT with restorative purpose is NOT NP in endodontics department.
B. Conditions of RP:
1. The condition of RP in this chart series is that a tooth has some pain, but no RCT or endo therapy was done or planned for the tooth.
2. Application of RCT or endo therapy to the tooth indicates that the tooth is NOT RP.
C. Conditions of AIP:
1. To be classified as AIP, the tooth needs to be described with all of the next conditions; deep caries, patient’s statements denying tooth pain, positive result of cold test or electric pulp test, and application or plan of RCT or endo therapy.
D. Conditions of SIP:
1. The cold test (+++) should be regarded as the primary evidence of SIP. A cold test (+) just means the pulp is vital. Just complaining of pain by the patient without a cold test does NOT constitute SIP evidence.
2. Pulpotomy, NOT pulpectomy, is one of temporary treatments for SIP.
E. Similarity and difference between PN and previous treatments:
1. The existence of apical abscess, apical periodontitis, apical pathosis indicates PN or previous treatment.
2. The pulp test result ‘Non vital’ indicates PN or previous treatment.
3. Apical periodontitis is a sign of PN or previous treatment.
4. The statements indicating continuation of treatments from previous visit, previously Initiated RCT, or ‘re-accessed’, are the clear indicators of previous treatment.
5. Existing temporary materials previously applied, including IRM (intermediate restorative material), cavit, or cotton, are the clear indicators of previous treatment.
6. Conversely, the use of such temporary materials at the current visit does NOT necessarily indicate previous treatment.
7. If a tooth showing the conditions of PN has previously treated root canal, it should be classified as ‘the others’.
(V) Important points for the tooth classification.
1. The existence of deep caries does NOT necessarily indicate pulpitis.
2. Performing RCT on a tooth does NOT necessarily mean that the tooth had deep caries, or that the pulp is non-vital or necrotic, or that pulp has irreversible pulpitis.
3. Asymptomatic apical periodontitis is NOT equal to AIP.
4. If obturation or gutta percha filling was performed at the first visit, the tooth is NOT SIP or PN.
5. Abbreviations, S:, O:, A:, P:, CC:, Dx, PI, Hx, Tx, Pt, HPI, NV:, EOE, IOE, PARL, EPT, PAP, SAP, NAT, RCT, BWX, PA, and NSRCT stand for Subjective, Objective, Assessment, Plan, Chief Complaint, Diagnosis, Previously Initiated, History, Treatment, Patient, History of Present Illness, Next Visit, Extra-Oral Examination, Intra-Oral Examination, Periapical Radiolucency, Electric Pulp Test, Primary Apical Periodontitis, Symptomatic or Secondary Apical Periodontitis, Normal Apical Test, Root Canal Treatment, Bite-Wing X-ray, Periapical (X-ray), and Non-Surgical Root Canal Therapy, respectively. UR, UL, LR, and LL indicate Upper Right, Upper Left, Lower Right, and Lower Left of quadrant of the mouth, respectively.

## Appendix C

Examples of chart interpretation/classification by the local LLM, only showing the charts diagnosed differently between human dentists and LLM on the end point of the randomly chosen 400-case trial (Result part, Section 3.1). Original chart notes were removed in order to protect patients’ information.

**Dentists**	**LLM**	**Reasoning by LLM**
Others	NP	The chart indicates the patient chose a root-canal treatment (“RCT/CL/PCC or RCT and core BU”) for tooth #3, which is an elective endodontic procedure. No pain, caries, or diagnostic findings of pulpitis are reported; thus, per rule IV A, this elective RCT is considered prophylactic and the pulp is classified as ** normal pulp (NP) **.
Others	AIP	The chart reports deep occlusal caries on tooth #18, a positive cold test (+), no pain reported, and normal periapical tissues. According to the decision rules, A = 3 (deep caries), B = 1 (no pain), and C = 2 (cold +) satisfy the criteria for asymptomatic irreversible pulpitis. The explicit diagnosis “normal pulp or asymptomatic irreversible pulpitis” also includes AIP as a possible outcome. No prior treatment or evidence of pulpal necrosis is present, so the tooth is classified as AIP.
Others	SIP	The chart explicitly diagnoses the tooth as “Pulpal: PI” (irreversible pulpitis). According to the instruction, any mention of irreversible or symptomatic pulpitis is classified as ** symptomatic irreversible pulpitis (SIP) **. The periapical finding (“asymptomatic apical periodontitis”) is used only as a check for pulpal necrosis and does not override the explicit pulpal diagnosis.
Others	SIP	The chart explicitly states “Treatment Recommended: RCT #3 with pulpectomy at evaluation visit for emergency care” following a chief complaint of severe pain. Performing a pulpectomy (root-canal therapy) on a tooth that is symptomatic and painful is the standard treatment for ** symptomatic irreversible pulpitis **; no other diagnosis is provided, so SIP is the appropriate classification.
Others	PN	The chart explicitly states “non-vital tooth” and records a completed root-canal treatment, indicating pulpal necrosis.
Others	SIP	The chart records the patient’s complaint of “terrible sensitivity” and current pain, and the clinician is performing a root-canal treatment on tooth #25. No explicit diagnosis is given, but the presence of symptomatic pain with an indicated RCT satisfies the definition of * symptomatic irreversible pulpitis * (SIP) per the provided rules.
AIP	Others	No explicit diagnostic statement was found in the chart. The tooth shows a pulp exposure, but there is no recorded pain, cold test, electric pulp test, or periapical radiolucency to apply the classification rules for NP, RP, AIP, SIP, or PN. Therefore, it is classified as “others”.
PN	Others	The chart contains no explicit diagnostic statement for tooth #11, and none of the required test results (cold/EPT) or pain findings is reported. While deep caries and a periapical radiolucency (PAP) are noted, the absence of a definitive pulp diagnosis and lack of cold-test evidence preclude classification as NP, RP, AIP, SIP, or PN. Therefore, the tooth is classified as “others”.
NP	AIP	The chart indicates a pulp exposure (A = 4), the patient is currently asymptomatic (B = 1), and the electric pulp test is positive (D = 2). According to rule III, these findings satisfy the criteria for ** asymptomatic irreversible pulpitis **. No explicit diagnosis or prior treatment evidence was present, so step I and II did not apply.
RP	Others	The chart lists an ambiguous diagnosis (“reversible pulpitis or SIP depending on carious excavation”) and no definitive evidence for any of the six defined categories. Cold test is + (not +++), and percussion pain is present, but there is no spontaneous pain or clear indication of irreversible pulpitis. Test-result criteria (A–F) do not match NP, RP, AIP, SIP, or PN, so the tooth is classified as “others”.
NP	Others	No explicit diagnostic statement for tooth #30 was found in the chart. The assessment mentions RCT on this tooth, but it is not described as elective or prophylactic, and no caries depth, pain description, pulp test results, or periapical radiolucency are provided. Consequently, none of the criteria for NP, RP, AIP, SIP, or PN can be satisfied, so the tooth is classified as “others”.
Other	SIP	The chart states that tooth #14 requires pulpotomy and RCT/PCC with crown, indicating symptomatic irreversible pulpitis. No prior treatment or test data are provided, but the presence of pain and the planned pulpotomy/RCF support a SIP diagnosis.
2	1	−1 NP
1	0	−1 RP
1	2	1 AIP
0	4	4 SIP
1	1	0 PN
7	4	−3 others

## Appendix D

**(Tables)** This appendix contains logistic regression analyzing the relationship between patients’ age and the frequencies of disease history, derived from the chart data used in main text. The outcomes in this study are the diagnosis of healthy (Y/N), cancer (Y/N), HIV (Y/N), and type 2 DM (Y/N). CI, confidence interval. **(Charts)** The chart under each table is a conditional estimates plot depicting the predicted association between patients’ age and each disease history indicated within the chart above. The bold curves indicate the modeled distribution of samples. The shadowed portions surrounding the curves indicate 95% CI areas. The dots at the top and bottom of each plot indicate actual standardized data.

**Healthy**					**Cancer**				
		**95% CI**					**95% CI**		
**Odds Ratio**	***p***	**Lower Bound**	**Upper Bound**	**McFadden R^2^**	**Odds Ratio**	***p***	**Lower Bound**	**Upper Bound**	**McFadden R^2^**
0.91	<0.001	0.900	0.913	0.300	1.08	<0.001	1.070	1.089	0.187

**HIV**					**Type 2 DM**				
		**95% CI**					**95% CI**		
**Odds Ratio**	***p***	**Lower Bound**	**Upper Bound**	**McFadden R^2^**	**Odds Ratio**	***p***	**Lower Bound**	**Upper Bound**	**McFadden R^2^**
1.03	<0.001	1.022	1.039	0.032	1.07	<0.001	1.06	1.073	0.154
